# Sub-classification of myopic glaucomatous eyes according to optic disc and peripapillary features

**DOI:** 10.1371/journal.pone.0181841

**Published:** 2017-07-24

**Authors:** Seungbong Han, Kyung Rim Sung, Jimin Park, Joo Young Yoon, Joong Won Shin

**Affiliations:** 1 Department of Applied Statistics, Gachon University, Seongnam-si, Korea; 2 Department of Ophthalmology, College of Medicine, University of Ulsan, Asan Medical Center, Seoul, Korea; 3 College of Medicine, University of Ulsan, Seoul, Korea; Bascom Palmer Eye Institute, UNITED STATES

## Abstract

**Purpose:**

To investigate the sub-classification of myopic glaucomatous eyes by optic disc and peripapillary features.

**Methods:**

Optic disc tilt and torsion were determined from retinal nerve fiber layer photographs. Based on the location of the Bruch’s membrane (BM) opening within the β-zone of the peripapillary atrophy (PPA) area, the widths of β-zone PPA (PPA1W), PPA_+BM_ (PPA2W), and PPA_-BM_ (PPA3W) were measured with enhanced depth imaging spectral-domain optical coherence tomography. Cluster analysis that employed partitioning around medoids was performed with these parameters, the presence of inward rotation of BM ending axial length (AXL), and central corneal thickness.

**Results:**

A total of 115 eyes (AXL≥24 mm) were included. Two clusters produced maximum overall silhouette widths (average = 0.43). Visual field (VF) mean deviation was not different between cluster 1 (52 eyes; -4.02±3.01 dB) and cluster 2 (63 eyes; -5.21±5.62 dB; p = 0.174). In cluster 1 compared to cluster 2, optic disc tilt was significantly greater, PPA1W and PPA3W were longer, and AXL was longer (all p<0.001). The presence of an inward rotation of BM ending was more frequent in cluster 2 (p = 0.043). Forty-one eyes (78.8%) in cluster 1 had superior VF defects while 10 eyes (19.2%) had inferior defects, and only one eye (2%) had defects in both hemifields. Eyes in cluster 2 were more evenly distributed: 55.6% had superior defects, 34.9% had inferior defects, and 9.5% had defects in both hemifields (p = 0.023).

**Conclusions:**

Myopic glaucomatous eyes characterized by optic disc and peripapillary configurations can be classified as two distinct types, and the most distinct difference between the two was degree of optic disc tilt and width of PPA. The location of VF defects were also significantly different between two clusters.

## Introduction

Myopia has been shown to be a risk factor for the development of glaucoma in many studies [1–9]. It can be postulated that the relationship between myopia and glaucoma is caused by mechanical stress. Myopic eyes have thinner sclera and lamina cribrosa (LC). Weaker LC may make myopic eyes more vulnerable to glaucomatous damage [10]. Additionally, myopic axial elongation may induce tensile stress on the optic disc and peripapillary area, which are the target tissues of glaucomatous structural damage. Tensile stress on the optic disc and peripapillary area from axial elongation may aggravate or mimic glaucomatous damage that occurs at the level of the LC. Accordingly, several publications have shown that the optic disc and peripapillary features found in myopic eyes are related to glaucomatous damage [11–15].

The stress and subsequent deformation of the optic disc and peripapillary area from axial elongation may be different among myopic eyes. Thus, glaucomatous changes can be differentially manifested according to myopic optic disc and peripapillary features. Hence, we hypothesized that there are subgroups of myopic glaucomatous eyes that have different features in the optic disc and peripapillary area that may have different clinical characteristics. If subclassification of myopic eyes according to those parameters related to optic disc and peripapillary area is possible, we may have some clue for clinical course of myopic glaucomatous eyes. Therefore, in this study, we tested our hypothesis that myopic glaucomatous eyes can be sub-classified according to the baseline features of the optic disc and peripapillary area that have been suggested to be related to glaucoma in previous publications [11–15]. Following sub-classification, we compared the clinical characteristics of the subgroups based on optic disc and peripapillary features.

## Methods

The medical records of all patients were evaluated by a single specialist (K.R.S.) at the glaucoma clinic of Asan Medical Center, Seoul, Korea, and baseline examinations were performed between March 2010 and May 2012. In this current analysis, only baseline data were evaluated. Baseline examinations included a comprehensive ophthalmologic examination, which included a review of medical history, measurement of best-corrected visual acuity (BCVA), slit-lamp biomicroscopy, multiple intraocular pressure (IOP) measurements using Goldmann applanation tonometry, gonioscopy, dilated fundoscopic examination using a 90- or 78-diopter (D) lens, stereoscopic optic disc photography, retinal nerve fiber layer (RNFL) photography, visual field (VF) testing, central corneal thickness (CCT) measurement (DGH-550, DGH Technology, Inc., Exton, PA), and axial length (AXL) measurement (IOL Master, Carl Zeiss Meditic, Inc. Dublin, CA). RNFL thickness measurements were performed using spectral-domain optical coherence tomography (OCT; SPECTRALIS OCT, Heidelberg Engineering, Dossenheim, Germany). Inclusion criteria were BCVA of 20/40 or better, AXL ≥ 24 mm, normal anterior chamber, and an open angle on slit-lamp and gonioscopic examinations. Glaucomatous optic disc changes were defined as diffuse or focal neural rim thinning, disc hemorrhage, and/or RNFL defects. Patients with any other ophthalmic or neurologic condition that could result in a VF defect, a history of diabetes mellitus, or severe myopic fundus precluding adequate examination were excluded. If both eyes of the patient were found to be eligible, one eye was randomly selected for analysis.

**Fig 1:**
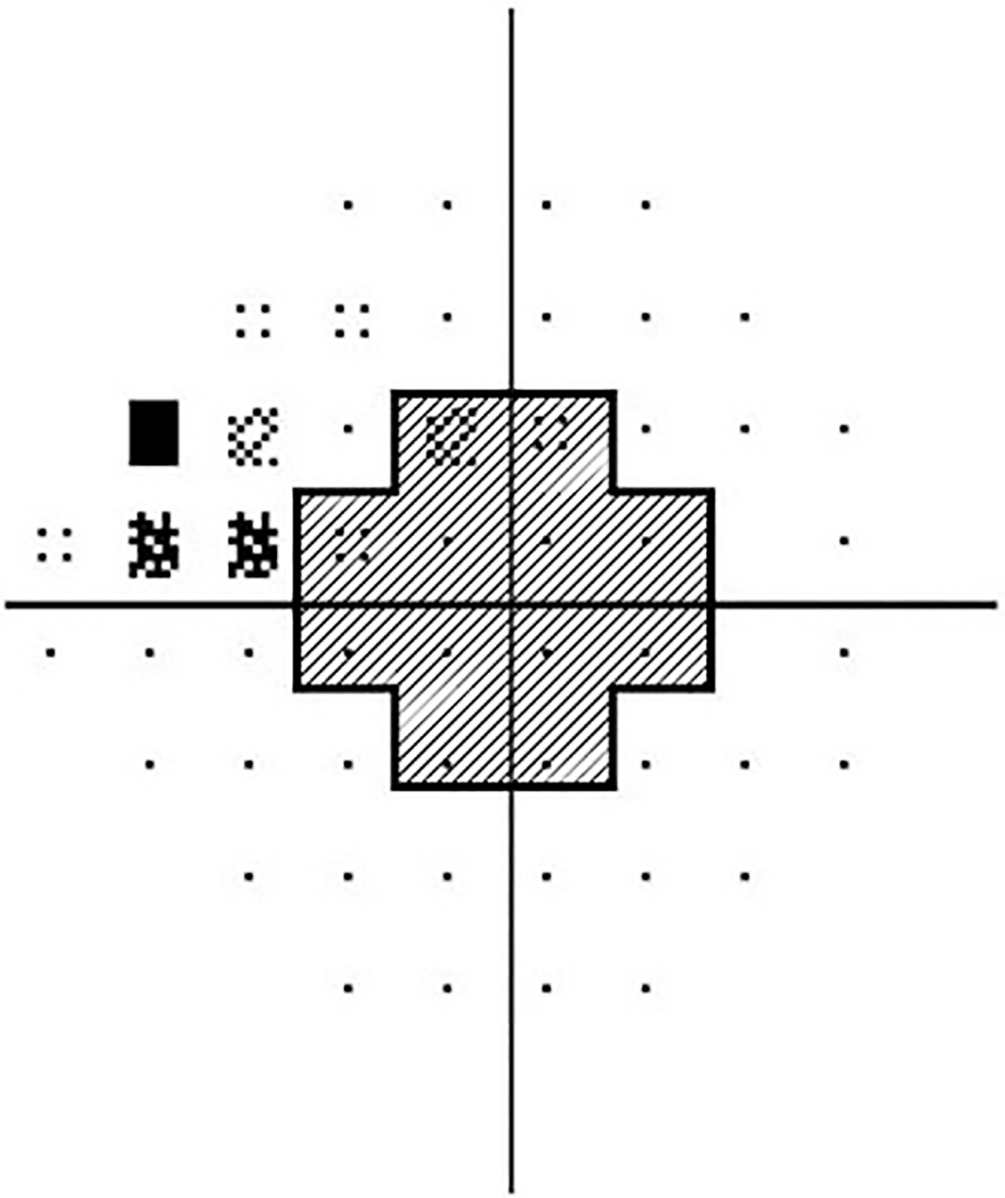
The central 10° region of the Humphrey 24–2 visual field (shaded area) was determined as illustrated. Two test locations within the blind spot and 10° to 24° regions were excluded.

VF tests were performed using a Humphrey field analyzer (Swedish Interactive Threshold Algorithm [SITA] 24–2; Carl Zeiss Meditec, Inc. Dublin, CA). Only reliable VF test results (false positive errors < 15%, false negative errors < 15%, and fixation loss < 20%) were included in the analysis. The VF test was repeated within two weeks of the baseline measurement for confirmation. Data from the first VF test were excluded to obviate any learning effects. All participants had to have glaucomatous optic disc and glaucomatous VF defects, as defined by glaucoma hemifield test results outside the normal limits or a pattern standard deviation outside the 95% range for normal limits. In addition, these eyes had to have a cluster of three points with probabilities < 5% on the pattern deviation map in ≥ one hemifield, including ≥ one point with a probability < 1% or a cluster of two points with a probability < 1%. Glaucomatous VF defects had to be confirmed on two VF examinations. To determine the location of VF defects, the 24–2 Humphrey visual field was divided into two areas within the central 10° region and 10–24° area (Fig 1). Eyes with clusters of three significant points with a probability less than 5% or a cluster of two points with a probability < 1% on the pattern deviation map within the central 10° were regarded as having a central VF defect and those outside the central 10° (thus in the 10–24°) were regarded as having a peripheral VF defect [16]. The location of VF defect clusters was then classified as present in the superior, inferior, or both hemifields. The institutional review board (IRB) of the Asan Medical Center approved the present study, informed consent was waived owing to the retrospective nature of the study, and the study design was executed in accordance with the principles of the Declaration of Helsinki. Data were collected by two authors (JP and JYY) and anonymized before analysis by one of the author (JWS).

### Assessment of optic disc tilt and torsion

**Fig 2:**
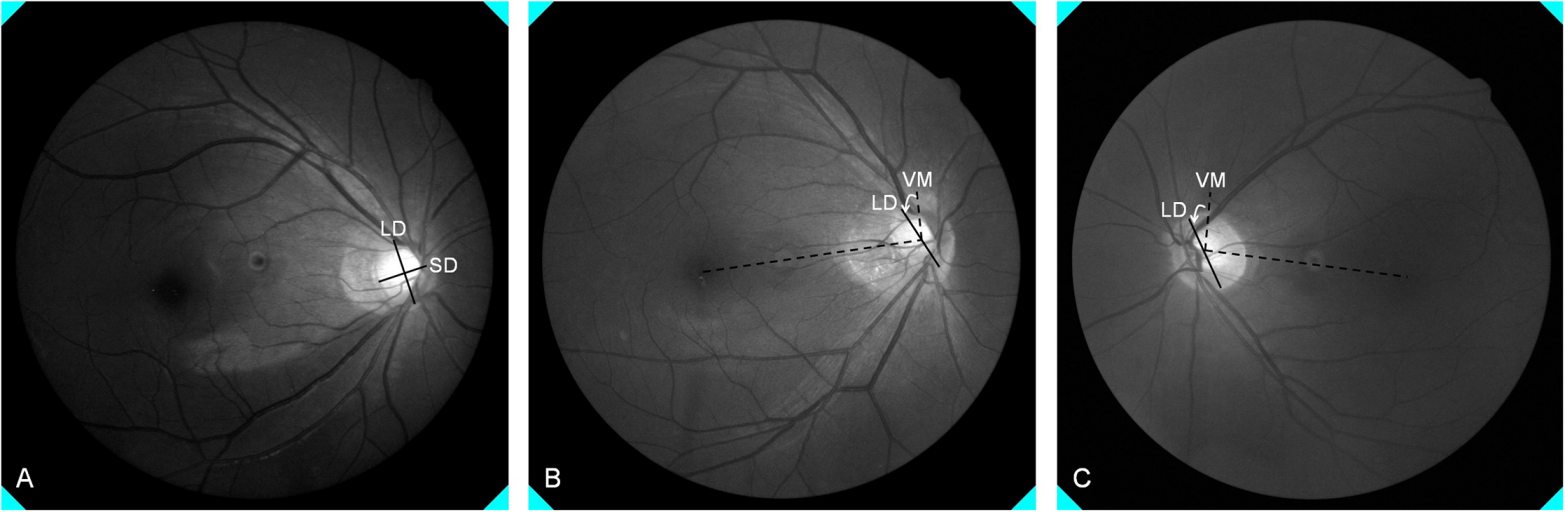
Optic disc tilt was identified using the ovality index, and the tilt ratio was defined as the ratio between the longest (LD) and shortest diameters (SD) of the optic disc (A). Optic disc torsion was defined as the deviation of the long axis of the optic disc from the vertical meridian (VM). The VM was identified as a vertical line 90° from a horizontal line connecting the fovea to the center of the optic disc. The angular degree between the long axis and VM of the optic disc was named the torsional degree. A positive torsion value indicated inferotemporal torsion (B), and a negative torsion value indicated superonasal torsion (C).

Red-free RNFL photographs centered on the optic disc were obtained using a non-mydriatic retinal camera. Optic disc tilt and torsion were measured on these photographs by a well-trained examiner (J.P) using ImageJ analysis software (ImageJ 1.48v, developed by Wayne Rasband, National Institutes of Health, Bethesda, MD). Optic disc tilt and torsion were defined according to previously described criteria [17, 18]. Optic disc tilt was identified using the ovality index. Hence, the tilt ratio was defined as the ratio between the longest and shortest diameters of the optic disc (Fig 2A). Optic disc torsion was defined as the deviation of the long axis of the optic disc from the vertical meridian (Fig 2B and 2C). The vertical meridian (VM) was identified as a vertical line 90° from a horizontal line connecting the fovea to the center of the optic disc. The angular degree between the long axis and VM of the optic disc was named the “torsional degree”. A positive torsion value indicated inferotemporal torsion (Fig 2B), and a negative torsion value indicated superonasal torsion (Fig 2C).

### Assessment of β-zone peripapillary atrophy (PPA) and Bruch’s membrane (BM)

**Fig 3:**
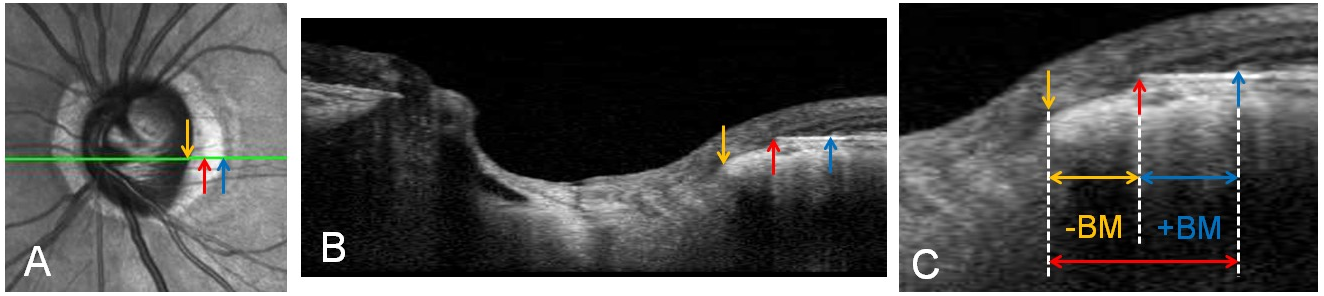
The temporal β-zone PPA margin (blue arrow), BM opening (red arrow), and disc margin (yellow arrow) were defined using infrared (IR) and B-scan images. The temporal disc margin and β-zone PPA margin was defined as the border between low and high reflectivity on IR images (A). The BM opening was identified on OCT B-scans as the termination of highly reflective continuous lines (B). On the basis of the location of BM opening within the β-zone PPA area, β-zone PPA was subdivided into PPA_+BM_, the zone from the β-zone PPA margin to BM opening, and PPA_-BM_, the zone from BM opening to the disc margin (C). The widths of β-zone PPA, PPA_+BM_, and PPA_-BM_ were measured on the IR images in synchronization with the OCT B-scan images. Mean β-zone PPA width (PPA1W), PPA_+BM_ width (PPA2W), and PPA_-BM_ width (PPA3W) of three B-scans were calculated.

**Fig 4:**
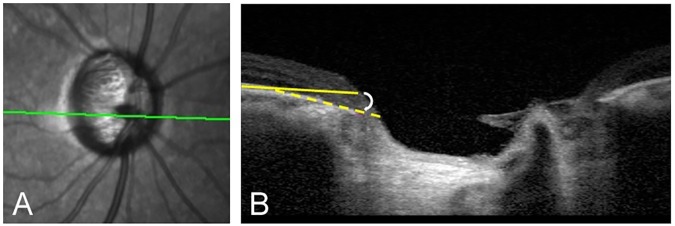
The presence of inward rotation of BM ending, defined as the angular degree between BM plane outside of a PPA region (yellow line) and BM plane within a PPA region (yellow dash line), was greater than 10°. Eyes were considered to have inward rotation of BM ending if it was found in at least one out of three B-scans (B).

Spectral-domain enhanced depth imaging (EDI) was used to scan the optic disc including the PPA area. Briefly, the entire optic disc was scanned using a 6-mm length line (512 A-scans) with an interval of 50μm. In our study, an average of 35 horizontal B-scans was produced in EDI mode. From these B-scans, three frames (center, mid-superior, and mid-inferior) that passed through the optic disc were selected [19]. The structure of the temporal β-zone PPA and optic disc was analyzed with the intrinsic viewer [12]. The temporal β-zone PPA margin, BM opening, and disc margin were defined using infrared (IR) and B-scan images by another examiner (J.Y.Y). The temporal disc margin and β-zone PPA margin was defined as the border between low and high reflectivity on IR images. The BM opening was identified on OCT B-scans as the termination of highly reflective continuous lines (Fig 3A–3C). Eyes were excluded when these points could not be clearly identified. On the basis of the location of BM opening within the β-zone PPA area, β-zone PPA was subdivided into PPA_+BM_, the zone from β-zone PPA margin to BM opening, and PPA_-BM_, the zone from BM opening to the disc margin. The widths of β-zone PPA, PPA_+BM_, and PPA_-BM_ were measured on the IR images in synchronization with the OCT B-scan images (Fig 3C). Mean β-zone PPA width (PPA1W), PPA_+BM_ width (PPA2W), and PPA_-BM_ width (PPA3W) of three B-scans were calculated. Additionally, we assessed the presence of inward rotation of BM ending, which was defined as the angular degree between BM plane of the outside of a PPA region in temporal side and BM plane within a PPA region was greater than 10 degree. If found in at least one out of three B-scans, those eyes were considered to have an inward rotation of BM ending (Fig 4A and 4B).

### Statistical analysis

Association between spherical equivalent and other parameters were assessed by Pearson correlation analysis. For subclassification of myopic glaucomatous eyes, we used a cluster analysis to classify myopic glaucomatous eyes into different groups based on the shape and characteristics of the optic disc and PPA. Cluster analysis is widely used to segment subjects into several subgroups in which subjects are closely related to each other. Although there are several clustering algorithms, we employed the partitioning around medoids (PAM) method [20]. PAM is more robust and less sensitive to outliers compared to the k-means clustering method. PAM is based on the search for *k* representative objects, called “medoids”, among observations. Here, the number of clusters *k* is pre-chosen. Then, *k* clusters are sequentially made by grouping each subject to the closest medoids. We aimed to minimize the sum of distances or dissimilarity between each observation to their corresponding medoid. It is imperative to select the correct number of clusters in the process of implementing PAM. We used a silhouette plot to determine *k*. We compared overall average silhouette widths for a predetermined *k* value. Large silhouette width indicates that the subject within the cluster is well classified. There are three optional metrics, “Euclidean”, “Manhattan”, or “Gower”, to define the dissimilarity [21]. We used the Euclidean metric, because it produced large silhouette widths. The clustering variables were as follows: presence of inward rotation of BM ending, torsion direction, disc tilt ratio, AXL, PPA widths (PA1W, PA2W, and PA3W), absolute torsion degree, and CCT. Following the cluster analysis, we compared variables describing the shape of the optic disc and peripapillary area and location of VF defect among clusters. Statistical significance was evaluated by the *t* test or Wilcoxon rank-sum test for continuous variables. For categorical variables, χ^2^ statistics or Fisher’s exact test were used. No adjustment was performed for multiple testing in several comparisons [22]. Statistical analyses were conducted using SPSS version 22.0 (SPSS Inc., Chicago, IL) and R software version 3.3.0 [23]. The R package “*cluster”* was used to implement the PAM analysis [24]. All reported P-values are two-sided, and P-values of less than 0.05 were considered to indicate statistical significance.

## Results

A total of 115 eyes were included in the final analysis. Fifty-nine were from men, and 56 were from women; the mean age was 50.8±12.4 years (range; 21~85) old. Overall, Optic disc and PPA parameters showed relationship with myopic degree determined by AXL (Table 1).

**Table 1:** Relationship between spherical equivalent and other parameters determined by Pearson correlation analysis.

	Correlation coefficient
Optic disc tilt ratio	0.443
PPA1W (micron)	0.267
PPA2W (micron)	-0.193
PPA3W (micron)	0.454
Absolute torsion degree (°)	-0.192

Abbreviations: PPA1W = β-zone peripapillary atrophy (PPA) width; PPA2W = β-zone PPA width with Bruch’s membrane (BM); PPA3W = β-zone PPA width without BM

**Fig 5:**
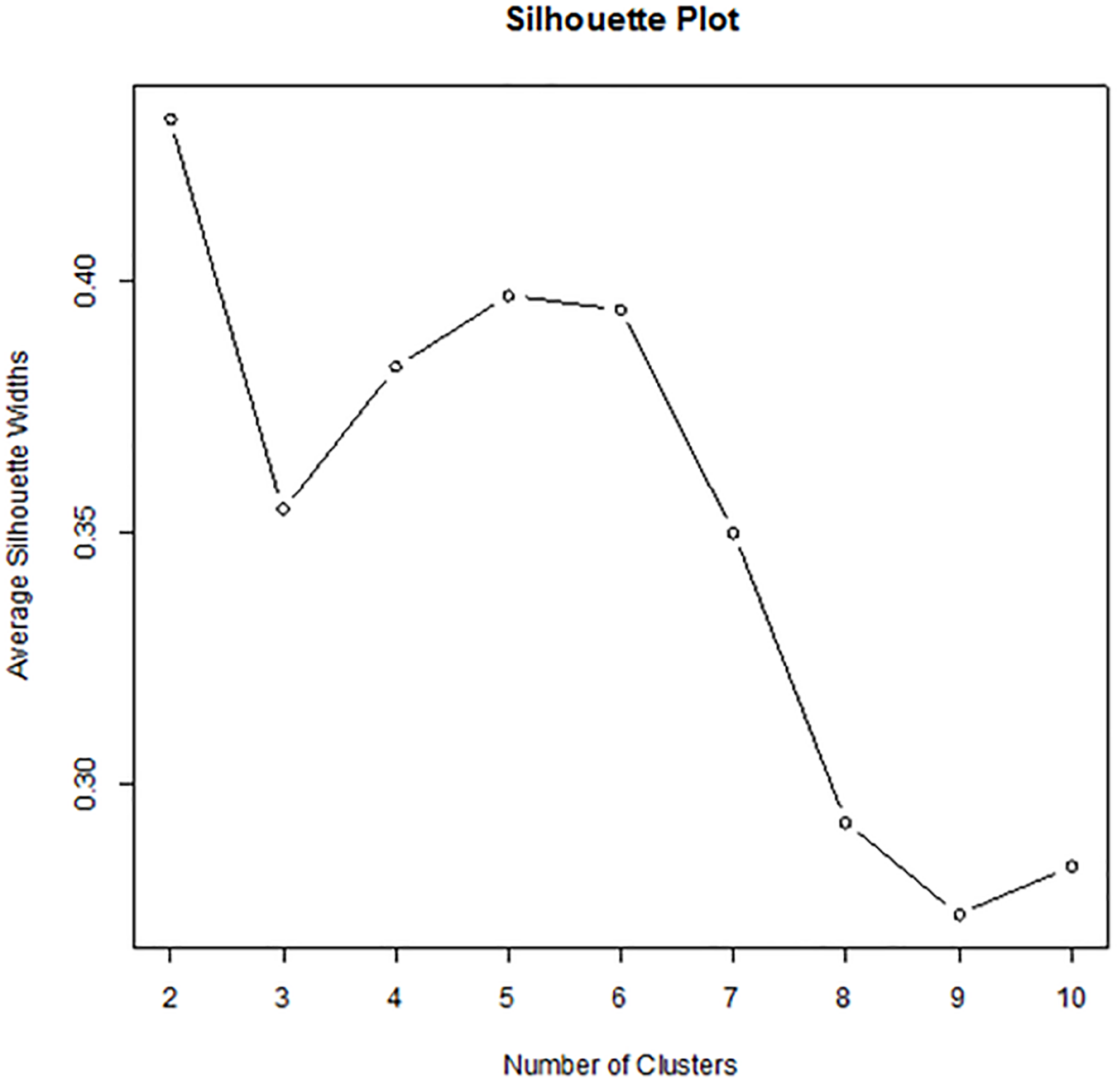
Overall average silhouette widths according to the number of clusters.

Fig 5 shows the overall average silhouette width according to the number of clusters. Two clusters (*k* = 2) produced the maximum overall silhouette width (average silhouette width = 0.43). Among 115 eyes, 52 eyes were classified as cluster 1, and 63 as cluster 2. Cluster 2 was more homogeneous than cluster 1 in that the silhouette widths of cluster 1 and 2 were 0.31 and 0.54, respectively.

When we compared demographics and baseline clinical characteristics, eyes in cluster 1 were younger than cluster 2 (48.0±11.9 versus 53.2±12.4 years, p = 0.024) and more myopic (-6.33±2.4 versus -4.4±1.9 diopters, p<0.001). However, other characteristics, such as gender proportion, baseline IOP, and glaucoma severity determined by either average RNFL thickness or VF mean deviation (MD) were not different between the two clusters. RNFL thickness was thinner at nasal and thicker at temporal quadrant in in cluster 1 (Table 2).

**Table 2:** Comparison of demographics and clinical characteristics of two subgroups determined by cluster analysis in myopic glaucomatous eyes.

	Cluster 1 (n = 52)	Cluster 2 (n = 63)	P value
Age (years)	48.0±11.9	53.2±12.4	0.024
Gender (M/F)	28/24	31/32	0.709
Baseline intraocular pressure (mmHg)	16.4±3.8	16.6±4.7	0.756
Spherical equivalent (diopter)	-6.3±2.4	-4.4±1.9	<0.001
Average RNFL thickness(micron) thickness ckness (micron)	76.3±13.3	75.3±16.1	0.747
Superior quadrant RNFL thickness (micron)	91.8±21.6	92.3±24.1	0.908
Inferior quadrant RNFL thickness (micron)	81.4±21.8	86.2±29.1	0.329
Nasal quadrant RNFL thickness (micron)	46.6±13.9	57.8±16.3	<0.001
Temporal quadrant RNFL thickness (micron)	78.9±17.3	63.3±16.5	<0.001
Visual field mean deviation (decibel)	-4.02±3.01	-5.21±5.62	0.174

Abbreviations: RNFL = retinal nerve fiber layer

When we compared optic disc and PPA features between the two clusters, optic disc tilt was significantly greater (p<0.001), PPA1W and PPA3W were longer (both, p<0.001), and AXL was longer in cluster 1 than cluster 2 (p<0.001; Table 3). The presence of inward rotation of BM ending was more frequent in cluster 2 (21.2% versus 34.9%, p = 0.043), while inferotemporal torsion of the optic disc was more frequent in cluster 1 (42.3% versus 63.5%, p = 0.026); however, PPA2W, absolute degree of torsion, and CCT were not different between the two clusters (Table 3).

**Table 3:** Comparison of optic disc and peripapillary area characteristics between two subgroups determined by cluster analysis in myopic glaucomatous eyes.

	Cluster 1 (n = 52)	Cluster 2 (n = 63)	P value
Optic disc tilt ratio	1.32±0.14	1.19±0.14	<0.001
Axial length (mm)	26.5±1.5	25.3±1.4	<0.001
PPA1W (micron)	608.7±152.7	322.2±108.6	<0.001
PPA2W (micron)	245.8±131.7	237.0±102.9	0.687
PPA3W (micron)	363.0±115.8	85.4±47.5	<0.001
Inferotemporal torsion	22 (42.3%)	40 (63.5%)	0.026
Absolute torsion degree (°)	16.2±12.3	17.8±13.5	0.516
Presence of inward rotation of BM	11 (21.2%)	25 (39.7%)	0.043
CCT (micron)	541.2±34.5	530.9±34.5	0.126

Abbreviations: BM = Bruch’s membrane; CCT = central corneal thickness; PPA1W = β-zone peripapillary atrophy (PPA) width; PPA2W = β-zone PPA width with BM; PPA3W = β-zone PPA width without BM

Comparing hemifield locations of VF defects between the two clusters, 78.8% of eyes in cluster 1 had a VF defect in the superior hemifield, 19.2% of eyes had a VF defect in the inferior hemifield, and only 2% of eyes (one eye) had a VF defect in both hemifields (Table 4). Moreover, eyes in cluster 2 were relatively evenly distributed with 55.6% showing VF defects in superior, 34.9% in inferior, and 9.5% in both hemifields (p = 0.023). Moreover, the two clusters showed significant differences in the presence of central VF defects (p = 0.007). Eyes classified as cluster 1 had peripheral VF defects more frequently than central VF defects (central, 11.5%; peripheral, 71.2%; both, 17.3%) compared to eyes in cluster 2 (central, 30.2%; peripheral, 42.9%; both, 27.0%).

**Table 4:** Comparison of visual field defect location between two subgroups determined by cluster analysis in myopic glaucomatous eyes.

Visual field location		Cluster 1 (n = 52)	Cluster 2 (n = 63)	P value
Hemifield	Superior	41 (78.8%)	35 (55.6%)	0.023
	Inferior	10 (19.2%)	22 (34.9%)	
	Both	1 (1.9%)	6 (9.5%)	
Central vs. peripheral	Central	6 (11.5%)	19 (30.2%)	0.007
	Peripheral	37 (71.2%)	27 (42.9%)	
	Both	9 (17.3%)	17 (27.0%)	

**Fig 6:**
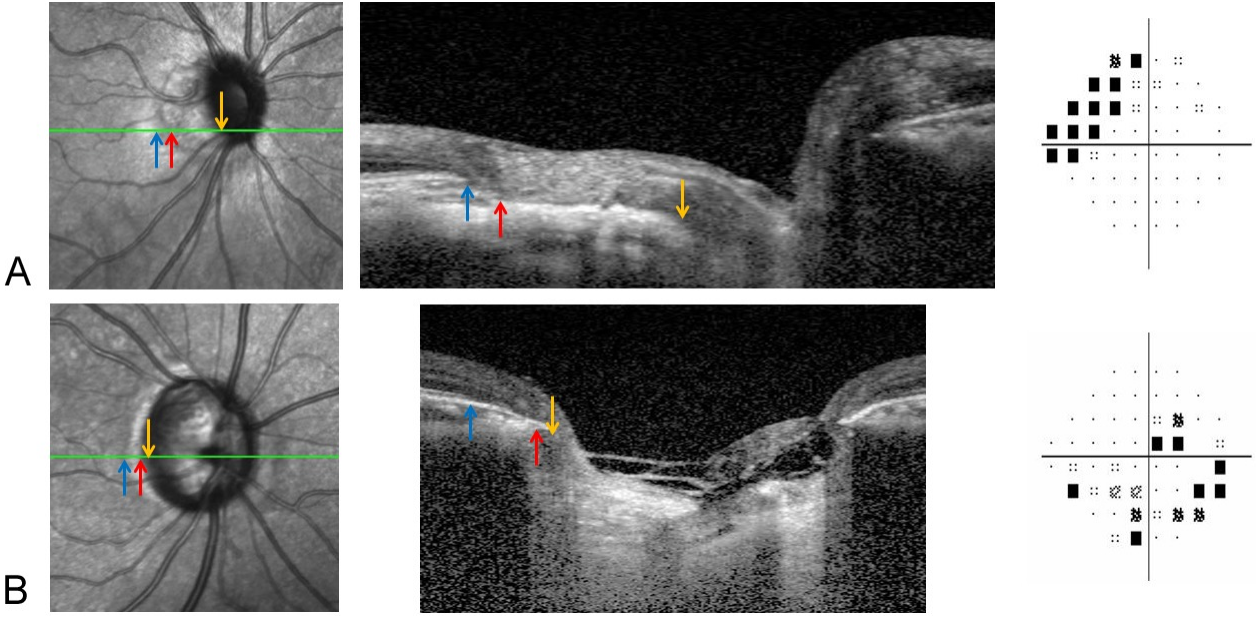
Representative clinical examples of two clusters. (A) Myopic glaucomatous eyes belonging to cluster 1 with greater optic disc tilt, longer total peripapillary atrophy width, and VF defects (mean deviation (MD); -5.81 dB) in the superior peripheral area. (B) Eyes belonging to cluster 2 with round-shaped optic discs with no tilt, inward rotation of BM ending, and VF defects (MD; -5.23 dB) in both hemifields including the central area.

Representative clinical examples are shown in Fig 6. Fig 6A shows the myopic glaucomatous eyes that belonged to cluster 1 with greater optic disc tilt, longer total PPA width, and VF defects (MD; -5.81 dB) in the superior peripheral area. Fig 6B shows eyes that belonged to cluster 2 with round-shaped optic discs without tilt, inward rotations of BM ending, and VF defects in both hemifields (MD; -5.43 dB), including the central area.

Sub-cluster analysis was performed with eyes belonged to cluster 2 (Table 5). Most prominent difference between two clusters (cluster 2 A and 2B) was optic disc torsion. All eyes in cluster 2A showed inferotemporal torsion while none of the eyes did in cluster 2B and absolute torsional degree was greater in cluster 2A. Comparing hemifield locations of VF defects between the two clusters, 50% of eyes in cluster 2A had a VF defect in the superior hemifield, 37.5% in the inferior hemifield, and 12.5% of eyes in both hemifields while 65.2%, 30.4% and 4.4% had a VF defect in superior, inferior and both hemifields in cluster 2B, respectively (p = 0.399). Presence of central VF defect were not different between cluster 2A and 2B (32.5% versus 26.1%, p = 0.521).

**Table 5:** Comparison of optic disc and peripapillary area characteristics between two subgroups belonged to cluster 2 determined by sub-cluster analysis in myopic glaucomatous eyes.

	Cluster 2A (n = 40)	Cluster 2B (n = 23) (n = 63)	P value
Optic disc tilt ratio	1.17±0.11	1.24±0.18	0.038
Axial length (mm)	25.0± 0.94	25.9± 1.87	0.076
PPA1W (micron)	316.1± 112.5	332.7± 103.2	0.436
PPA2W (micron)	236.7± 113.0	237.5± 84.8	0.617
PPA3W (micron)	79.8± 46.3	95.2± 49.1	0.119
Inferotemporal torsion	40 (100%)	0 (0%)	<0.001
Absolute torsion degree (°)	21.3± 14.1	11.7± 9.9	0.002
Presence of inward rotation of BM	16 (40%)	9 (39.1%)	1.000
CCT (micron)	533.3± 32.0	527.0± 38.6	0.503

Abbreviations: BM = Bruch’s membrane; CCT = central corneal thickness; PPA1W = β-zone peripapillary atrophy (PPA) width; PPA2W = β-zone PPA width with BM; PPA3W = β-zone PPA width without BM

## Discussion

Our results showed that myopic glaucomatous eyes could be clearly separated into two clusters based on optic disc and PPA features. The two clusters had different anatomical characteristics in the optic disc and peripapillary area. Among 115 eyes, 52 eyes (45.2%) were classified as cluster 1, and 63 eyes (54.8%) were classified as cluster 2. Cluster 1 eyes had longer AXL, wider PPA, and greater degree of optic disc tilt. Hence, this group of patients had more pronounced alterations in the optic disc and PPA according to myopic axial elongation. Although optic disc tilt and presence of PPA are considered hallmarks of myopic optic discs, more than half of myopic glaucomatous eyes that belonged to cluster 2 (54.8%) did not have substantial alterations in their optic discs (mean ovality index, 1.19). Eyes in cluster 1 were from significantly younger subjects compared to those from cluster 2, but other demographic and clinical characteristics, such as baseline IOP or glaucoma severity determined by average RNFL thickness or VF MD, were not significantly different between the two clusters.

When we compared optic disc and PPA parameters, most of them were substantially different between the two clusters. In cluster 1, total PPA width and PPA width not including BM were significantly wider, and optic tilt was greater. However, PPA width including BM was not different between the two clusters. An interesting finding was that the inward rotation of BM ending was significantly more frequent in cluster 2. Hence, although PPA width with BM was not different between the two clusters, the configuration of BM ending was different between the two clusters. The clinical significance of the inward rotation of BM has not been investigated previously, but we assume that if BM ending moves posteriorly, LC of the optic disc may also displace posteriorly; thus, optic disc cupping would be increased, which may, theoretically, worsen glaucomatous axonal damage.

Previous reports have suggested that optic disc tilt during myopization may induce stretching of the nerve fiber and thus damage the RNFL; this would account for why myopic glaucomatous eyes with optic disc tilt does not progress more quickly in terms of glaucoma than that of eyes without optic disc tilt, as myopization slows when the patient reaches adulthood [15, 25–27]. Taking this into account, eyes in cluster 1 may have been affected more by myopic tensile changes in the RNFL, while eyes in cluster 2 may have been influenced more by optic disc cupping in the RNFL.

Optic disc tilt is reported to be caused by stretching of the peripapillary sclera on one side of the optic disc [15]. If 360 degrees of the peripapillary sclera is evenly stretched; the optic disc would have a round rather than tilted shape. Hence, it may enable the eyes belonging to cluster 1 were more likely to have peripapillary scleral thinning on one side of the optic disc, affecting tensile strength in one direction. Such unidirectional tension and increased likelihood of inferior torsion may enable superior VF defects. Moreover, eyes in cluster 2 showed symmetrical stretching of the peripapillary sclera in all directions or minimal peripapillary scleral stretching and had relatively round-shaped optic discs without tilt (mean ovality index, 1.19) along with substantially decreased total PPA width (PPA1W) compared to cluster 1 eyes. Instead, BM ending of eyes belonging to cluster 2 underwent inward rotation more frequently, which suggested that more posterior displacement of LC may occur. Glaucoma severity assessed by both RNFL thickness and VF MD was not different between the two clusters. However, the locations of VF defects were significantly different between the two groups with similar levels of overall VF MD. The majority of eyes that belonged to cluster 1 had VF defects only in the superior hemifield, however, eyes in cluster 2 were relatively evenly distributed between both superior and inferior hemifield VF defects; additionally, eyes that had VF defects in both hemifields were more frequently found in cluster 2. This may be connected to the observation that inferotemporal torsion was more frequently found in cluster 1. Hence, eyes in cluster 1 may be more seriously affected by optic disc and peripapillary changes due to axial elongation, and subsequently, VF damage in these eyes followed the direction of change. Eyes in cluster 2, which are suspected to be especially affected by pure glaucomatous changes, had more evenly distributed VF defects throughout the hemifields. We also investigated the possibility that eyes belonged to cluster 2 was further subdivided. Sub-cluster analysis revealed eyes belonged to cluster 2 was classified according to the direction of optic disc torsion and degree of absolute torsion, but those two sub-clusters did not show difference in terms of VF defect pattern.

In conclusion, our analysis revealed that myopic glaucomatous eyes had two distinct types characterized by optic disc and peripapillary changes. More than half of myopic glaucomatous eyes lacked serious alterations in their optic disc shape or PPA, but these eyes had more frequent changes in their BM opening endings, suggesting that posterior shifting of LC may be accelerated in these optic discs. These two groups had different profiles in their VF presentation; damages were either relatively evenly manifested or concentrated in the superior hemifield. Such observations suggest that some myopic glaucomatous eyes were influenced by both myopic changes and glaucomatous damage while others were more influenced by glaucomatous damage. This notion may be more intensely discussed in our forthcoming analysis incorporating longitudinal data.

## Supporting information

S1 File This is the raw data file. (CSV)
